# Ulcers

**Published:** 1890-10

**Authors:** 


ULCERS.—Kali-bichrom: ulcers become deeper without spreading. They look as if cut with a punch. Nitric acid deep ulcers—especially chancres—with indurated edges.

